# Synthesis of novel (1-alkanoyloxy-4-alkanoylaminobutylidene)-1,1-bisphosphonic acid derivatives

**DOI:** 10.1186/1860-5397-2-2

**Published:** 2006-02-24

**Authors:** Petri A Turhanen, Jouko J Vepsäläinen

**Affiliations:** 1 University of Kuopio, Department of Chemistry, P.O. Box 1627, FIN-70211, Kuopio, Finland

## Abstract

A novel strategy for the synthesis of (1-alkanoyloxy-4-alkanoylaminobutylidene)-1,1-bisphosphonic acid derivatives (**1a-d**) via (1-hydroxy-4-alkanoylaminobutylidene)-1,1-bisphosphonic acid derivatives (**2a-d**), starting from alendronate has been developed with reasonable 51–77% overall yields. Intermediate products, (1-hydroxy-4-alkanoylaminobutylidene)-1,1-bisphosphonic acid derivatives (**2a-d**), were prepared in water with reasonable to high yields (52–94%).

## Introduction

**Figure 1:**
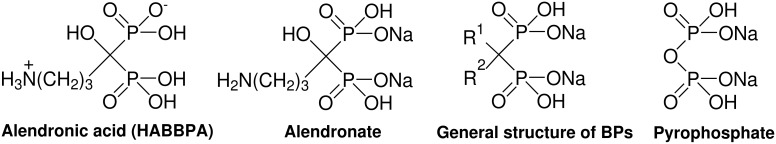


Bisphosphonates (BPs) are analogs of naturally occurring pyrophosphate and are used as drugs for the treatment of various bone diseases, like osteoporosis. [1–5] Alendronate, (1-hydroxy-4-aminobutylidene)-1,1-bisphosphonic acid (HABBPA) disodium salt is a typical example of an amino BP compound (Figure 1). Traditionally BPs have been used for decades as drugs for the treatment of various bone diseases but recently these compounds have been found to be active in many other fields, such as against parasitic diseases [6–10] and atherosclerosis. [11] Unfortunately BPs are very hydrophilic, highly anionic and their bioavailabilities are very poor. [12–14] It would be a clear advance to prepare more lipophilic and biodegradable derivatives of BPs. A rather straightforward method to improve the lipophilicity of amino BPs is to convert hydroxyl or/and amino group or the phosphonic acid groups with a biodegradable ester or amide functionality.

Our group has designed, synthesized and studied *in vitro* several different clodronate (R^1^ = R^2^ = Cl, Figure 1) and etidronate (R^1^ = CH_3_ R^2^ = OH, Figure 1) ester and amide derivatives to act as biodegradable prodrugs. [15–19] We have also recently described the method for the preparation of (1-alkoxycarbonyloxyethylidene)-1,1-bisphosphonic acid derivatives [20] and novel fatty acid derivatives of etidronate [21] which may represent prodrugs of etidronate. In the case of etidronic acid, we have shown that simple phosphonate esters like P(O)-O-Me as well as an acyl group at the tertiary alcohol group are stable against enzymatic hydrolysis. [18] However, the latter decomposed gradually due to chemical hydrolysis.

Because of the extreme hydrophilic character of HABBPA, its solubility and reactivity in organic solvents is very low. As far as we are aware there are only six known examples of amide (R-CO-HN-) derivatives of commercially used aminobisphosphonates (alendronate and pamidronate) in the literature, [22–24] but the hydroxyl derivatives are unknown. Probably one reason for this is the above mentioned solubility and reactivity problems.

## Results and discussion

**Scheme 1:**
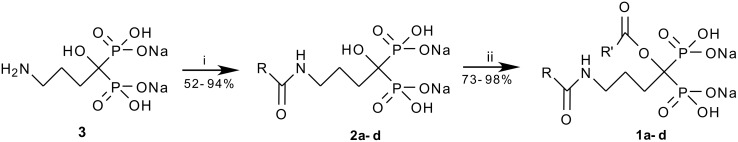
Preparation of (1-alkanoyloxy-4-alkanoylaminobutylidene)-1,1-bisphosphonic acid derivatives **1a** (R = R' = Me), **1b** (R = R' = Et), **1c** (R = Pr; R' = Me) and **1d** (R = Bu^t^; R' = Me). Conditions: i) (RCO)_2_O in H_2_O; ii) (R'CO)_2_O then H_2_O (if necessary).

Here we report the first method to prepare (1-alkanoyloxy-4-alkanoylaminobutylidene)-1,1-bisphosphonic acid derivatives **1a-d** via (1-hydroxy-4-alkanoylaminobutylidene)-1,1-bisphosphonic acid compounds **2a-d** (see Scheme 1). The preparation of the intermediate compounds **2a**-**d** was rather straightforward and was started from the mono sodium salt of HABBPA which was prepared and isolated as previously reported. [25] HABBPA mono sodium salt (200 mg) was stirred in water (2 ml) for a few minutes before a 40% NaOH solution (78 μl, 1.0 eq of NaOH) was added and the mixture was stirred until a clear solution was obtained. After addition of anhydride (1 ml), the mixture was stirred overnight (ca. 16 h for **2a-b** and 24 h for **2c-d**) and water was evaporated *in vacuo*, diethyl ether (10–15 ml) was added and the white precipitate was filtered and dried *in vacuo*. Since the products of **2a-d** contained a few percent of the sodium salt of the corresponding carboxylic acid formed in the reaction and in the case of **2c**, the reaction mixture contained 31% of starting material **3** (reaction did not proceed to completion in even 3 days) according to ^1^H and ^31^P NMR spectra, they required purification. Furthermore, in the case of **2a** and **2b**, 9% and 7% of **1a** and **1b**, respectively, were formed in the reaction. Compounds **2a-b** were purified by dissolving in H_2_O (0.5 ml) and adding dry MeOH (3.5 ml) by stirring before the mixtures were placed in the freezer for 30 minutes. Solids were filtered and washed with H_2_O/MeOH (1:7) and dried *in vacuo* to produce **2a** and **2b** with 82% and 81% yields, respectively. Compound **2c** was purified (if needed) by stirring in absolute EtOH for 30 minutes (this was repeated twice if necessary) to give **2c** with 94% yield. Compound **2d** was dissolved in water (3 ml) and dry MeOH (4 ml) was added by stirring before the mixture was placed in the freezer for weekend. The mixture was filtered and the filtrate was evaporated to dryness *in vacuo* to give **2d** with a 52% yield. Alendronic acid (HABBPA), which was synthesized as reported earlier [25] (except for the isolation procedure, since in our hands, no precipitation occurred), was also tested as the starting material for the preparation of **2a** (its acid form), but no reaction was observed under the test conditions.

This is not an unexpected result since alendronic acid, which has pK_a_ values of 0.8, 2.2, 6.3, 10.9 and 12.2 (Ezra *et al*. [26]), exists as a zwitterion (see Figure 1) and the ammonium group is protected against electrophilic attack of the acyl group.

Also the mono sodium salt of alendronic acid was a poor starting material due to the same reason but ca. 37% of **2a** was obtained. As expected, the *in situ* prepared disodium salt of HABBPA was a good starting material because of its good solubility in water [solubility of HABBPA, its mono- and disodium salts in water (5 ml) were 41.4 mg, 161.7 mg and > 1000 mg, respectively, which were tested in room temperature] and reactivity of amino group when an excess of anhydride was used.

Since the amine group of alendronic acid is protected due to zwitterion, we attempted to acetylate the middle carbon OH-group directly by using alendronic acid and its monosodium salt under dry conditions, as we did for etidronic acid, [27] but unfortunately this did not prove feasible. However, acetylation was successful with this method when starting with the disodium salt of HABBPA. However, this led to a diacetylated product, with an acetyl moiety both, at the amino and the hydroxyl group.

Based on this finding the target compounds **1a-d** were prepared from **2a-d** using this method by stirring in an excess of acetic anhydride (**1a,c,d**) or propionic anhydride (**1b**). In the case of **1a** and **1c**, the reaction time was only 2 h and 8 h, respectively, whereas the reaction time for **1b** and **1d** was 48 h. We also tried to react compound **2c** with butyric anhydride but the reaction did not occur under the test conditions. Acylation of OH-group with trimethylacetic anhydride was not successful due to steric hindrance of the trimethylacetic group. The target compounds **1a-d** were isolated by filtration after addition of diethyl ether (10–15 ml) to the reaction mixture or by washing the residue with diethyl ether after the reaction mixture was evaporated to dryness. If needed, after drying the products were dissolved in a small volume of water and stirred for 40 minutes at room temperature for hydrolyzing any of formed bisphosphonate dimers which were obtained as determined by the ^31^P NMR spectra. Compounds **1a-c** were purified like **2c** by stirring in absolute EtOH, compound **1d** needed no purification steps. All compounds (**1a-d**) still contained some sodium salt of the corresponding carboxylic acid (≤ 5%).

The formation of the intermediates **2a-d** and the target compounds **1a-d** during the reactions was readily confirmed from ^1^H and ^31^P NMR spectra. In ^31^P NMR spectra, the signals shift to the higher ppm value after the amino group had been acylated [e.g. for **3** from 18.59 ppm to 18.98 ppm (**2a**)]. However, after acylation of the OH-group, the ^31^P NMR signals shift to the lower ppm value [e.g. for **2a** from 18.98 ppm to 14.65 ppm (**1a**)] as expected from the earlier results of the OH-group acylation from etidronic acid derivatives. [18,20,27]

## Conclusion

In conclusion, the first synthesis of novel (1-alkanoyloxy-4-alkanoylaminobutylidene)-1,1-bisphosphonic acid derivatives (**1a-d**) have been reported with good overall yields (51–77%) starting from alendronate. (1-Hydroxy-4-alkanoylaminobutylidene)-1,1-bisphosphonic acid derivatives (**2a-d**) were prepared and isolated with 52–94% yields as intermediate product for synthesis of **1a-d**. All of the prepared compounds **1a-d** and **2a-d** are proposed to be more lipophilic and are thus more soluble in organic solvents than alendronate and can be used as starting materials for the synthesis of new phosphorus end modified derivatives of HABBPA and which may represent as possible prodrugs of alendronate. Novel biological evaluation for selected compounds of **1a-d** and **2a-d** will be carried out and published in future.

## Supporting Information

File 1 Experimental procedures and full spectroscopic data for all new compounds.
