# Supplementary material for: Synthesis of novel (1-alkanoyloxy-4-alkanoylaminobutylidene)-1,1-bisphosphonic acid derivatives
Source: Beilstein J Org Chem. 2006 Feb 24;2:2. doi: 10.1186/1860-5397-2-2 (PMC1408082; doi:10.1186/1860-5397-2-2)
Supplement: File 1 — Experimental procedures and full spectroscopic data for all new compounds. [file Beilstein_J_Org_Chem-02-02-s001.doc]

Synthesis of novel (1-alkanoyloxy-4-alkanoylaminobutylidene)-1,1-bisphosphonic acid derivatives

Petri A. Turhanen^*^ and Jouko J. Vepsäläinen

# Additional material:

# Experimental

^1^H, ^31^P and ^13^C NMR spectra were recorded on a Bruker Avance 500 spectrometer operating at 500.1, 202.5 and 125.8 MHz, respectively. TSP was used as an internal standard for ^1^H and ^13^C measurements, and 85% H_3_PO_4_ was used as an external standard for ^31^P measurements. The ^n^*J_HP_* couplings were calculated from proton spectra and all *J* values are given in Hz. The number of protons on each carbon were detected from DEPT-135 experiments and marked after each carbon by the letters d (doublet), t (triplet), q (quartet) or qv (quintet). The *^n^J_CP_* couplings were calculated from carbon spectra with the coupling constants given in parenthesis as hertz. The purity of the products was determined from ^1^H and ^31^P NMR spectra and was ≥ 95 % if not stated otherwise. Electronspray ionization mass spectra were acquired by an LCQ quadrupole ion trap mass spectrometer with an electronspray ionization source (Finnigan MAT, San Jose, CA) using negative mode. All the prepared compounds (**2a-d** and **1a-d**) were not melted (or decomposed) at even 310 ˚C, so the exact melting points were not reasonable to measure.

**Procedure for the preparation of 2a-d:** Alendronic acid mono sodium salt (200 mg, 0.74 mmol) was suspended in H_2_O (2 ml), 40% NaOH solution in H_2_O (78 µl, 1.0 eq of NaOH) was added and the mixture was stirred as long as any solids were dissolved. Carboxylic acid anhydride (1 ml) was added and the reaction mixture was stirred for 16 h (**2a-b**) or 24 h (**2c-d**) at 60 °C before H_2_O was removed *in vacuo* and ether (10-15 ml) was added with stirring. Solids were filtered, washed with H_2_O/MeOH (1:7) (**2a-b**) or with abs. EtOH (**2c**) and dried *in vacuo*. **2d** was purified as reported earlier in this article. Products were present as a white powder with 52-94% yields after purification. Compound **2a** purity was ca. 93% containing ca. 7% of compound **1a**.

**Procedure for the preparation of 1a-d:** Compounds **2a-d** were stirred in excess of acetic anhydride (**2a,c,d**) or propionic anhydride (**2b**) for 2, 48, 8 and 48 h, respectively, at 60 °C. The reaction mixture was evaporated to dryness *in vacuo* and residue was washed with ether; or ether (ca. 10-15 ml) was added to the reaction mixture with stirring before the reaction mixture was placed in the freezer for 0.5 h and filtered. Solids were dried *in vacuo*, before being dissolved in H_2_O and stirred for ca. 40 minutes to hydrolyze formed dimers if needed. **1a-c** were purified by stirring in abs. EtOH for 30 minutes (which was repeated twice if necessary). No purification was needed for **1d**. The products were present as a white powder with 73-98% yields.

**(1-acetyloxy-4-acetylaminobutylidene)-1,1-bisphosphonic acid disodium salt** (**1a**)**:** ^1^H NMR (D_2_O): δ 3.19 (t, 2H, *J* = 6.8), 2.24 (m, 2H), 2.12 (s), 1.98 (s), 1.78 (m, 2H). ^13^C NMR (D_2_O) δ 180.20 s, 175.76 (t, ^3^*J*_CP_ = 5.0), 86.17 (t, ^1^*J*_CP_ = 129.0), 42.72 t, 33.59 t, 26.71 (tt, ^2^*J*_CP_ = 6.1), 24.85 q, 24.10 q. ^31^P NMR (D_2_O) δ 14.65. ESI-MS: 332.0 (M + 1).

**(1-propionyloxy-4-propionylaminobutylidene)-1,1-bisphosphonic acid disodium salt** (**1b**)**:** ^1^H NMR (D_2_O): δ 3.19 (t, 2H, *J* = 6.8), 2.43 (q, 2H, *J* = 7.6), 2.25 (m, 2H), 2.24 (q, *J* = 7.7), 1.77 (m, 2H), 1.104 (t, 3H, *J* = 7.7), 1.096 (t, 3H, *J* = 7.6). ^13^C NMR (D_2_O) δ 180.93 s, 178.95 (t, ^3^*J*_CP_ = 4.9), 85.83 (t, ^1^*J*_CP_ = 128.8), 42.58 t, 33.60 t, 32.16 t, 30.84 t, 26.28 (tt, ^2^*J*_CP_ = 6.2), 12.60 q, 11.43 q. ^31^P NMR (D_2_O) δ 14.72. ESI-MS: 360.0 (M + 1).

**(1-acetyloxy-4-butyrylaminobutylidene)-1,1-bisphosphonic acid disodium salt** (**1c**)**:** ^1^H NMR (D_2_O): δ 3.21 (t, 2H, *J* = 6.8), 2.25 (m, 2H), 2.21 (t, 2H, *J* = 7.4), 2.12 (s), 1.80 (m, 2H), 1.60 (tq, 2H, *J* = 7.4), 0.90 (t, 3H, *J* = 7.4). ^13^C NMR (D_2_O) δ 179.96 s, 175.75 (t, ^3^*J*_CP_ = 5.0), 86.14 (t, ^1^*J*_CP_ = 128.4), 42.54 t, 40.68 t, 33.65 t, 26.77 (tt, ^2^*J*_CP_ = 6.0), 24.06 q, 21.92 q, 15.69 q. ^31^P NMR (D_2_O) δ 14.65. ESI-MS: 360.0 (M + 1).

**(1-acetyloxy-4-pivaloylaminobutylidene)-1,1-bisphosphonic acid disodium salt** (**1d**)**:** ^1^H NMR (D_2_O): δ = 3.22 (t, 2H, *J* = 6.8), 2.24 (m, 2H), 2.13 (s), 1.78 (m, 2H), 1.18 (s). ^13^C NMR (D_2_O) δ 185.29 s, 175.70 (t, ^3^*J*_CP_ = 5.1), 86.15 (t, ^1^*J*_CP_ = 128.7), 42.58 t, 41.25 s, 33.46 t, 29.46 q, 26.94 (tt, ^2^*J*_CP_ = 6.0), 24.05 q. ^31^P NMR (D_2_O) δ 14.73. ESI-MS: 374.0 (M + 1).

**(1-hydroxy-4-acetylaminobutylidene)-1,1-bisphosphonic acid disodium salt** (**2a**)**:** ^1^H NMR (D_2_O): δ = 3.20 (t, 2H, *J* = 6.7), 1.99 (s), 1.94 (m, 2H), 1.82 (m, 2H). ^13^C NMR (D_2_O) δ 176.96 s, 76.73 (t, ^1^*J*_CP_ = 133.9), 42.94 t, 33.94 t, 26.21 (tt, ^2^*J*_CP_ = 6.4), 24.76 q. ^31^P NMR (D_2_O) δ 18.98. ESI-MS: 290.1 (M + 1).

**(1-hydroxy-4-propionylaminobutylidene)-1,1-bisphosphonic acid disodium salt** (**2b**)**:** ^1^H NMR (D_2_O): δ 3.21 (t, 2H, *J* = 6.8), 2.26 (q, 2H, *J* = 7.7), 1.96 (m, 2H), 1.82 (m, 2H), 1.11 (t, 3H, *J* = 7.7). ^13^C NMR (D_2_O) δ 180.89 s, 76.73 (t, ^1^*J*_CP_ = 134.0), 42.84 t, 33.93 t, 32.06 t, 26.29 (tt, ^2^*J*_CP_ = 6.4), 12.50 q. ^31^P NMR (D_2_O) δ 18.97. ESI-MS: 304.0 (M + 1).

**(1-hydroxy-4-butyrylaminobutylidene)-1,1-bisphosphonic acid disodium salt** (**2c**)**:** ^1^H NMR (D_2_O): δ 3.21 (t, 2H, *J* = 6.9), 2.22 (t, 2H, *J* = 7.4), 1.96 (m, 2H), 1.82 (m, 2H), 1.60 (tq, 2H, *J* = 7.4), 0.91 (t, 3H, *J* = 7.4). ^13^C NMR (D_2_O) δ 179.97 s, 76.73 (t, ^1^*J*_CP_ = 134.3), 42.84 t, 40.63 t, 33.97 t, 26.28 (tt, ^2^*J*_CP_ = 6.5), 21.89 q, 15.65 q. ^31^P NMR (D_2_O) δ 18.97. ESI-MS: 318.1 (M + 1).

**(1-hydroxy-4-pivaloylaminobutylidene)-1,1-bisphosphonic acid disodium salt** (**2d**)**:** ^1^H NMR (D_2_O): δ 3.22 (t, 2H, *J* = 6.9), 1.95 (m, 2H), 1.81 (m, 2H), 1.18 (s, 9H). ^13^C NMR (D_2_O) δ 185.29 s, 76.74 (t, ^1^*J*_CP_ = 133.3), 43.00 t, 41.24 s, 33.94 t, 29.44 q, 26.54 (tt, ^2^*J*_CP_ = 6.2). ^31^P NMR (D_2_O) δ 19.03. ESI-MS: 332.1 (M + 1).
